# The Role of Intravascular Imaging in Coronary Chronic Total Occlusion PCI: Enhancing Procedural Success Through Real-Time Visualization

**DOI:** 10.3390/jpm15070318

**Published:** 2025-07-15

**Authors:** Hussein Sliman, Rim Kasem Ali Sliman, Paul Knaapen, Alex Nap, Grzegorz Sobieszek, Maksymilian P. Opolski

**Affiliations:** 1 Technion Israel Institute of Technology, Haifa 3436212, Israel; dr.hussein.sliman@gmail.com; 2 Heart Center, Department of Cardiology, Carmel Medical Center, Haifa 3436212, Israel; 3 Department of Cardiology, Amsterdam UMC, University of Amsterdam, Amsterdam Cardiovascular Sciences, 1105 AZ Amsterdam, The Netherlands; p.knaapen@amsterdamumc.nl (P.K.); a.nap@amsterdamumc.nl (A.N.); 4 Department of Pediatrics, Clalit Health Care Organization, Carmel Medical Center, Haifa 3436212, Israel; 5 Wojskowy Szpital Kliniczny z Polikliniką SP ZOZ w Lublinie, 20-049 Lublin, Poland; grzes.bies@interia.pl; 6 Department of Interventional Cardiology and Angiology, National Institute of Cardiology, Alpejska 42, 04-628 Warsaw, Poland; opolski.mp@gmail.com

**Keywords:** chronic total occlusion (CTO), percutaneous coronary intervention (PCI), intravascular ultrasound (IVUS), optical coherence tomography (OCT), coronary computed tomography angiography (CCTA)

## Abstract

Coronary chronic total occlusions (CTOs) are diagnosed in a significant portion of patients undergoing coronary angiography and represent one of the most complex scenarios in contemporary percutaneous coronary interventions (PCI). This review systematically examines how adjunctive imaging modalities’—intravascular ultrasound (IVUS), optical coherence tomography (OCT), and coronary computed tomography angiography (CCTA)—co-registration enhances the precision and success rates of CTO-PCI during the procedure. The strategic integration of these technologies enables the development of patient-specific intervention strategies tailored to individual vascular architecture and lesion characteristics. This personalized approach marks a transition from standardized protocols to precision interventional cardiology, potentially optimizing procedural success rates while minimizing complications.

## 1. Introduction

Although chronic total occlusions (CTOs) are prevalent in up to 35% of patients undergoing coronary angiography and often induce refractory angina, percutaneous revascularization attempts remain low (10–22%), with a wide variability between sites [1]. This conservative approach stems from concerns about clinical benefits, historically low success rates, potentially higher complication rates of CTO percutaneous coronary intervention (PCI) compared to the PCI of subtotal stenoses, and longer procedural times. However, recent advancements in techniques, equipment, and imaging modalities have improved CTO PCI outcomes, with expert operators now achieving success rates of around 90% [2]. Consequently, a CTO PCI has become a viable option for several indications, according to current guidelines from the European Society of Cardiology (ESC) and the American Heart Association (AHA); a CTO-PCI is indicated for patients with persistent symptoms (such as angina or dyspnea) despite optimal medical therapy or in selected patients with a large area of documented ischemia in the territory of the occluded vessel, provided that the anticipated benefits outweigh the procedural risks and the likelihood of success is high [3,4]. However, the decision should always involve a careful risk–benefit assessment, considering the procedural complexity, patient comorbidities, and operator/center expertise.

Conventional angiography, although foundational, provides a limited two-dimensional visualization that can be insufficient for navigating complex CTOs. The focus of this review is specifically on imaging techniques used during the CTO-PCI procedure itself. While pre-procedural imaging is valuable for case selection and strategy planning, intraprocedural imaging provides immediate, real-time guidance that directly impacts technical success and safety. IVUS and OCT offer a detailed visualization of the internal vessel, guiding the wire navigation, confirming the true lumen position, and optimizing stent deployment. Meanwhile, the CCTA co-registration with fluoroscopy provides an enhanced spatial orientation during complex interventions. When applied during the intervention, these complementary imaging modalities enable operators to make evidence-based decisions in real-time, adapting their approach based on precise anatomical information that conventional angiography alone cannot provide.

This review examines the evidence for intraprocedural imaging modalities in CTO PCI, analyzing their impact on procedural success and clinical outcomes to guide optimal imaging selection.

## 2. Intravascular Ultrasound

Intravascular ultrasound (IVUS) is a catheter-based imaging modality that has emerged as a crucial adjunct to angiography for guiding percutaneous coronary interventions (PCI) [5,6]. Its axial resolution of 100–150 μm and the ability to visualize both the lumen and the vessel wall, as well as the reference dimensions, stenosis severity, lesion length and morphology, and plaque burden, are crucial for procedural planning and device selection. Furthermore, it provides a detailed visualization of the spatial relationship between the coronary guidewire and the true lumen of the CTO. It aids guidewire navigation by clearly delineating the true lumen, subintimal space, and side branches, enhancing the operator precision and safety during occlusion crossing [7,8]. Available IVUS probes in the market include mechanical and solid-state (phased array) systems. Mechanical IVUS probes, such as the OptiCross™ (Boston Scientific, Marlborough, MA, USA) and Eagle Eye Platinum (Philips Amsterdam, Netherlands), offer a higher resolution (40–45 MHz). Solid-state systems, such as the Volcano IVUS (Philips), operate at lower frequencies (20 MHz) but provide better penetration. Newer probes, such as the AnteOwl WR (Terumo Corp, Tokyo, Japan), have a shorter distance between their tip and sensor, making navigation through complicated, blocked arteries easier [9,10,11,12].

### 2.1. IVUS Applications in the Antegrade Approach

IVUS enhances antegrade CTO PCI in three key areas: resolving proximal cap ambiguity, evaluating the guidewire position, and facilitating antegrade dissection and re-entry.

- **Proximal cap puncture**

Resolving proximal cap ambiguity is crucial for successful CTO crossing. Conventional angiography often fails to precisely identify the CTO entry point, risking a guidewire misdirection into the extraplaque space and potential procedural failure [13,14,15]. The IVUS-guided proximal cap puncture provides a precise intraluminal navigation of the CTO guidewire, which is particularly beneficial in stumpless CTO lesions. This enhanced visualization significantly improves the likelihood of successful wire crossing and reduces complications associated with an improper entry into the CTO [13,14,15,16,17].

**Figure 1:**
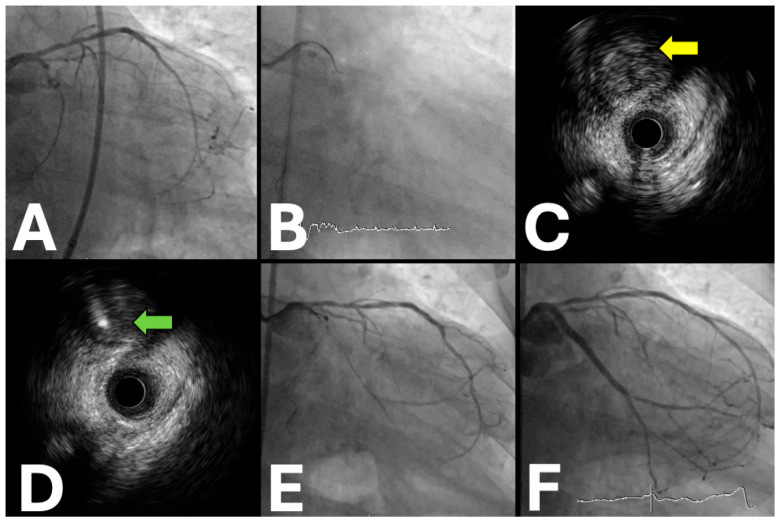
IVUS-guided proximal cap puncture technique in circumflex CTO intervention. (**A**) Baseline angiography demonstrating complete proximal circumflex occlusion with ambiguous entry point. (**B**) Fluoroscopic visualization during real-time IVUS guidance for optimal wire positioning. (**C**) IVUS cross-section revealing non-calcified proximal cap morphology ideal for puncture (yellow arrow). (**D**) IVUS confirmation of successful intraplaque wire penetration with optimal central positioning (green arrow). (**E**) Angiographic result following successful recanalization with restored vessel flow. (**F**) Final result after drug-eluting stent deployment, achieving excellent angiographic outcome.

The IVUS-guided proximal cap puncture is a key technique for resolving proximal cap ambiguity in CTO procedures [18,19,20,21]. This method involves advancing the IVUS probe to the side branch and carefully withdrawing it to identify the proximal cap location. Selecting an appropriately sized side branch, free from significant angulations, ostial stenosis, or calcification, is crucial for both the diagnostic and uncomplicated accommodation of the IVUS probe [20,22,23]. Moreover, IVUS aids in understanding the plaque morphology and guiding the wire selection for effective cap penetration. In cases with a side branch located at the CTO’s proximal end, calcium is often located opposite the side branch take-off, potentially complicating the guidewire penetration. Two approaches for IVUS-guided proximal cap puncture exist; the first is real-time guidance, where the IVUS probe is continuously positioned to optimize the stump visualization, allowing for the real-time monitoring of the guidewire position and ensuring a successful puncture, ideally in the central region of the stump [18]. This method requires large guiding catheters to accommodate both the IVUS catheter and the microcatheter—such as 8-F for Corsair Pro (Asahi Intecc), Turnpike Spiral (Teleflex), or Mamba (Boston Scientific) or 7-F for Finecross (Terumo Corp.), Caravel, Corsair Pro XS (Asahi Intecc), and Turnpike LP (Teleflex). The potential limitation of a real-time IVUS-guided proximal cap puncture relies on the wire and microcatheter deflection away from the stump, particularly in the case of a side branch with a shallow angle to the occluded vessel [20]. The second method uses IVUS to identify the CTO entry site, then withdraws to perform a tension-free proximal cap puncture. The IVUS re-advancement after the penetration confirms the intraluminal guidewire positioning [23,24], with reported success rates of 81% [25,26]. Both techniques enhance the CTO intervention success by providing real-time anatomical guidance during the guidewire manipulation (Figure 1).

- **Antegrade wiring**

**Figure 2:**
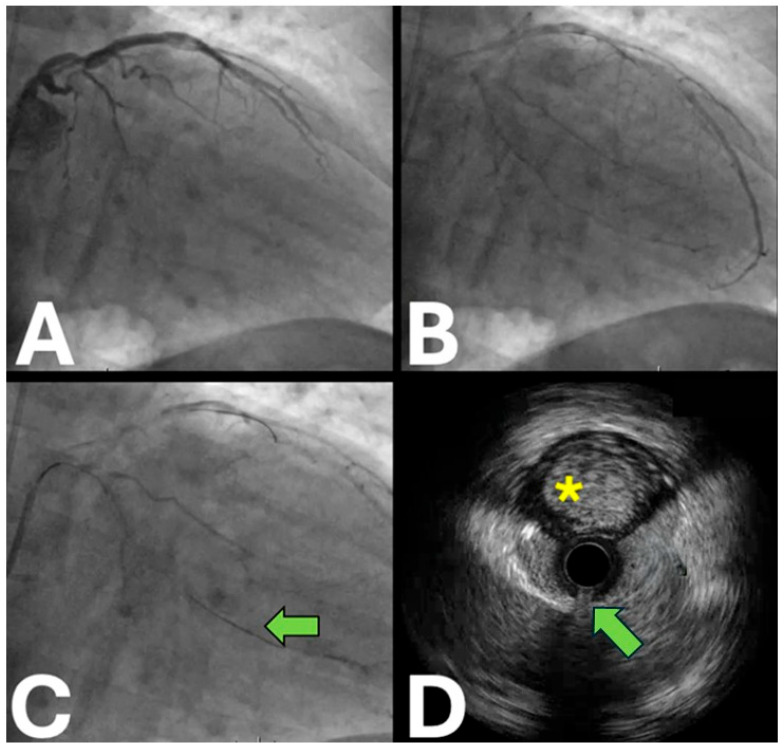
Intravascular ultrasound for demonstrating the extraplaque guidewire position. (**A**,**B**) The baseline coronary angiography of a chronic total occlusion of the proximal circumflex artery. (**C**) The coronary angiography displays the position of the intermediate tip load polymeric guidewire along the distal segment of the circumflex artery. (**D**) Intravascular ultrasound confirming the extraplaque guidewire position (green arrow) with a large lumen at 12 o’clock (yellow asterisk).

In CTO procedures, precise guidewire positioning is crucial for successful outcomes. While a contralateral injection is the primary method for confirming the wire location during antegrade CTO PCI, IVUS proves invaluable when distinguishing between the distal true lumen and the extraplaque guidewire location in challenging cases with a hampered angiographic visualization [9]. IVUS enhances the safety and efficacy of antegrade wiring attempts by clearly visualizing the wire position, allowing operators to navigate a complex CTO anatomy with greater confidence and precision (Figure 2).

- **Antegrade dissection and re-entry**

**Figure 3:**
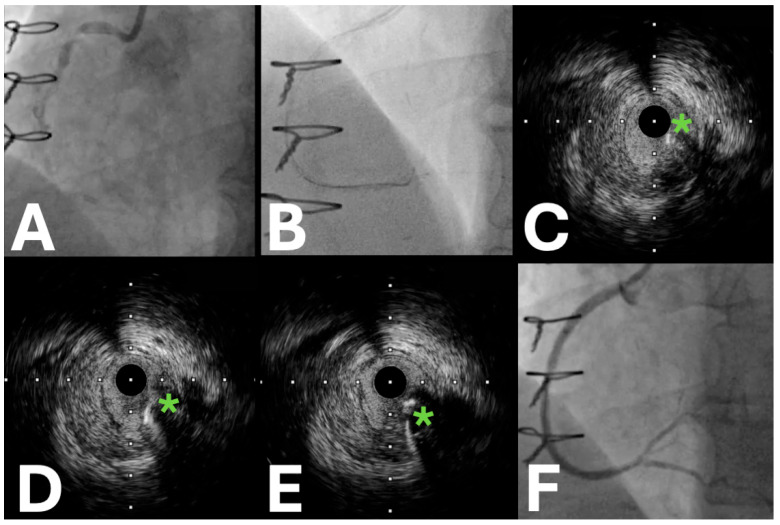
Intravascular ultrasound is used for the tip detection, antegrade dissection, and re-entry. (**A**) The baseline coronary angiography of a chronic total occlusion of the proximal right coronary artery. (**B**) The fluoroscopic image of the tip detection–antegrade dissection and re-entry under intravascular ultrasound guidance. (**C**) The shaft of the stiff guidewire on the intravascular ultrasound. (* indicates the guidewire position) (**D**,**E**) The tip of the stiff guidewire entering the true lumen on the intravascular ultrasound. (* indicates the guidewire tip position). (**F**) The final angiographic result after the stent implantation.

The antegrade dissection and re-entry (ADR) technique is particularly effective for long and complex occlusions where conventional re-entry methods may be insufficient. It involves an intentional subintimal entry, controlled dissection, and re-entry into the true lumen using dedicated devices like the Stingray balloon (Boston Scientific) or IVUS guidance. Alternative strategies for distal re-entry include the use of dual-lumen microcatheters or the TD-ADR (tip detection antegrade and dissection) technique [21]. The latter technique employs IVUS guidance for real-time guidewire navigation into the true lumen. Unlike conventional IVUS-guided wiring, TD-ADR utilizes continuous pullback to differentiate the guidewire tip from the shaft, enabling a precise perpendicular puncture into the distal CTO true lumen [26,27,28,29,30] (Figure 3).

The IVUS insertion into the extraplaque space may require small balloon predilatation; contrast injections are contraindicated. A stiff-tip guidewire with a high penetration force is recommended for successful re-entry. IVUS can guide a second wire into the distal true lumen when the initial wire enters a side branch beyond the occlusion. This approach enhances the precision and safety in challenging CTO interventions.

### 2.2. IVUS Applications in the Retrograde Approach

IVUS provides valuable guidance in retrograde CTO procedures, particularly during retrograde guidewire crossing and reverse controlled antegrade retrograde tracking (CART) techniques [21].

- **Retrograde wiring**

In ostial occlusions or bifurcations with blunt stumps, IVUS offers a visual confirmation of the retrograde guidewire position and re-entry into the true lumen. This visualization helps prevent serious complications, such as an aortic dissection or left main injury in ostial occlusions of the left anterior descending or the left circumflex arteries. Adjusting the position of the retrograde guidewire with the assistance of IVUS ensures a safer approach in these critical scenarios.

For complex scenarios, including bifurcations with blunt stumps and/or intra-stent CTOs after a failed antegrade recanalization, IVUS-guided retrograde approaches often prove useful. Side branch IVUS positioning enables the clear visualization of the retrograde guidewire at the CTO entry point, facilitating the re-entry into the true lumen within stent struts. IVUS also serves as an effective bail-out strategy when antegrade wires go subintimal, confirming the retrograde wire position in the proximal true lumen and enhancing the procedural safety and success.

- **Reverse controlled antegrade retrograde tracking**

**Figure 4:**
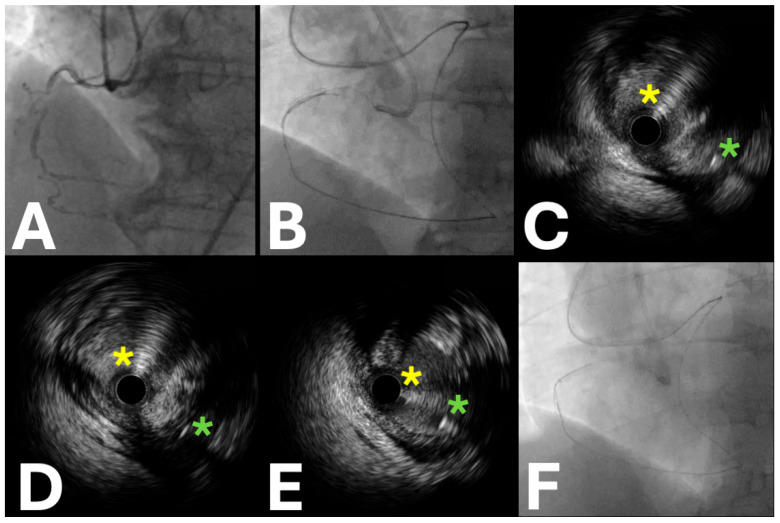
Intravascular ultrasound is used to enhance the tracking of difficult reverse controlled antegrade and retrograde procedures. (**A**) The baseline coronary angiography of a chronic total occlusion of the mid-right coronary artery. (**B**) The fluoroscopic image of intravascular ultrasound-guided reverse controlled antegrade and retrograde tracking. (**C**,**D**). Intravascular ultrasound displaying the intraplaque position of the antegrade wire (yellow asterisk) and the extraplaque position of the retrograde wire (green asterisk)—guidewires in different spaces. (**E**) Intravascular ultrasound showing both the antegrade wire (yellow asterisk) and the retrograde wire (green asterisk) in the intraplaque space, pinpointing the most convenient spot for reverse controlled antegrade and retrograde tracking. (**F**) The successful re-entry of the retrograde wire into the antegrade guide extension based on intravascular ultrasound guidance.

Reverse controlled antegrade retrograde tracking (reverse-CART) is characterized by antegrade subintimal dilation to connect antegrade and retrograde guidewires. IVUS plays a pivotal role in guiding difficult reverse CART procedures (with its different subtypes such as “conventional,” “directed,” “extended,” “mother-and-child-assisted,” “stent-assisted”, etc.), particularly when intraplaque tracking with the retrograde wire fails. Even after a 1:1 antegrade balloon dilatation, the connection may be impossible if guidewires occupy different spaces (intraplaque or extraplaque) [31]. IVUS determines precise wire positions, identifies optimal connection sites, locates less calcified segments for reentry attempts, and guides the balloon sizing for medial disruption, enabling the safer navigation of challenging occlusions (Figure 4).

## 3. IVUS for Lesion Preparation and Stenting in CTO PCI

The CTO PCI represents a significant challenge, with higher restenosis and re-occlusion risks compared to the PCI of subtotal stenoses [32,33]. Stent failure, often resulting from stent undersizing and/or under-expansion in chronically under-perfused vessels, remains a significant concern. On the contrary, oversized stents in negatively remodeled CTO segments risk dissection or perforation. IVUS serves as a crucial adjunctive tool for optimal stent selection, ensuring an appropriate length and diameter to minimize acute and long-term stent failure risks [34,35,36,37].

Recent meta-analyses have demonstrated that an IVUS-guided drug-eluting stent (DES) implantation outperforms angiography-guided percutaneous coronary interventions (PCI), particularly in complex lesions. This approach is associated with superior outcomes, notably reducing major adverse cardiac events (MACEs), including future myocardial infarction and stent thrombosis [38,39]. Furthermore, IVUS ensures optimal PCI results by confirming complete lesion coverage, adequate stent expansion and apposition, and the absence of significant stent edge dissections, thereby reducing the risks of restenosis and thrombosis while minimizing contrast use. The IVUS integration in CTO PCI enhances decision-making, improves stent selection, and optimizes procedural outcomes.

### 3.1. Clinical Data

IVUS-guided stent implantation demonstrates superior clinical outcomes compared to angiography-guided procedures. Early research showed that IVUS-guided DES implantation reduces in-stent restenosis and stent thrombosis [40,41]. Subsequent studies consistently reported improved clinical outcomes in challenging scenarios such as long lesions, acute coronary syndrome, and complex bifurcations [42,43,44]. Recent meta-analyses highlighted that IVUS guidance in complex lesions after a DES implantation leads to fewer MACEs, primarily due to the reduced target lesion revascularization [25,41].

Multiple randomized trials demonstrate significant benefits of IVUS guidance in CTO-PCI, including reduced MACE rates (2.6% vs. 7.1%), lower restenosis rates (3.9% vs. 13.7%), and decreased stent thrombosis (0.9% vs. 6.1%) compared to angiography-guided procedures [22]. These results highlight the ability of IVUS to optimize the stent placement and reduce complications [45,46,47]. Ongoing trials investigating the value of intravascular imaging in CTO PCI are presented in Table 1.

### 3.2. Critical Evidence Analysis and Controversies in IVUS-Guided CTO Interventions

While randomized trials demonstrate the benefits of IVUS in CTO-PCI, significant methodological concerns exist regarding the generalizability of these findings to routine clinical practice. The CTO-IVUS and AIR-CTO trials enrolled only 402 and 230 patients, respectively, at expert centers with experienced operators, raising questions about their applicability in community hospitals with varying operator expertise [39,43]. The selection bias compounds these limitations, as most IVUS studies exclude patients with renal dysfunction, severe calcification, or hemodynamic instability, precisely the high-risk populations where imaging guidance might provide the greatest clinical benefit. Procedural efficiency remains a topic of controversy, with proponents arguing that IVUS reduces the procedure time through improved navigation, while critics note that the imaging setup, acquisition, and interpretation can add 15–30 min to complex procedures, potentially offsetting the benefits of navigation.

Economic disputes persist, as IVUS catheters cost USD 500–USD 800 per case, but no definitive cost-effectiveness analyses exist comparing long-term clinical benefits against immediate procedural costs and potential complications from extended procedure times. Operator dependency concerns further complicate implementation, as IVUS interpretation requires substantial experience. Studies have shown significant inter-operator variability in image analysis and clinical decision-making, suggesting that this learning curve limitation may restrict benefits to high-volume centers and potentially exacerbate healthcare disparities between academic and community practice settings.

### 3.3. Future Implications

Ongoing research and technological advancements are needed to address the current limitations of IVUS in CTO PCI. Developing a forward-looking IVUS, improved calcification visualization, and enhanced integration with other imaging modalities could further optimize CTO PCI procedures. Additionally, standardized and artificial-enhanced protocols for image interpretation could improve the adoption and effectiveness of IVUS-guided techniques. Addressing these challenges will likely make IVUS even more valuable for improving CTO PCI patient outcomes.

## 4. Optical Coherence Tomography (OCT)

OCT offers complementary capabilities to IVUS in CTO interventions through its superior resolution (10–20 μm vs. 100–150 μm) despite its limited tissue penetration (1–2 mm vs. 4–8 mm). This light-based imaging technique offers a detailed visualization of the vessel wall and the characterization of plaque, making it particularly advantageous for stent optimization and post-procedural evaluation.

### 4.1. Practical Utility

While IVUS has been more extensively studied in CTO PCI, OCT provides distinct advantages through its superior resolution and microstructural visualization. This near-infrared light-based technology offers an exceptional clarity in differentiating plaque compositions (fibrous, calcified, and lipid-rich) within occluded segments. OCT particularly excels in the precise assessment of stent deployment characteristics, including the strut apposition, expansion, and tissue coverage—critical factors in preventing stent-related complications in complex CTO interventions. Its ability to detect subtle abnormalities, such as malposition, edge dissections, and early thrombosis formation, provides interventionalists with detailed insights that potentially enhance procedural safety and long-term outcomes, despite the additional contrast requirements for optimal image acquisition.

### 4.2. Clinical Data

OCT excels in characterizing CTO mechanisms, particularly in in-stent CTOs, differentiating between neointimal hyperplasia, stent under-expansion, and neoatherosclerosis to guide mechanism-specific interventions. In the RENOVATE-COMPLEX-PCI trial, OCT-guided procedures accounted for 20% of intravascular imaging cases [42].

### 4.3. OCT Versus IVUS: Clinical Controversies and Evidence Gaps

OCT faces significant limitations in CTO interventions that challenge its widespread adoption compared to IVUS. OCT’s limited penetration depth of 1–2 mm may inadequately assess vessel walls in heavily calcified or large-diameter vessels, potentially missing critical anatomical information that IVUS provides with its superior penetration of 4–8 mm. Contrast-related safety concerns arise as OCT requires multiple injections for blood clearance, raising safety issues in patients with renal dysfunction and potentially exacerbating dissections during complex CTO interventions.

Unlike IVUS, OCT lacks dedicated randomized trials in CTO populations, with most evidence deriving from subset analyses of broader studies. The RENOVATE-COMPLEX-PCI trial included only 20% OCT-guided procedures, severely limiting the statistical power for CTO-specific conclusions [42]. Timing controversies persist as some operators prefer OCT for post-intervention optimization due to its superior resolution, while others argue that IVUS provides more comprehensive guidance throughout the entire procedure. The choice remains controversial, as there are no evidence-based guidelines for modality selection in specific CTO scenarios, leading to an inconsistent clinical application and limiting the development of standardized protocols.

### 4.4. Future Implications

As research continues, the role of OCT in optimizing CTO PCI outcomes may become more clearly defined, potentially leading to a broader adoption and improved patient care. Future studies comparing OCT directly to IVUS in CTO PCI could provide valuable insights into the relative merits of these imaging modalities in this challenging subset of coronary interventions.

## 5. Coronary Computed Tomography Angiography (CCTA)

### 5.1. Introduction

Coronary computed tomography angiography (CCTA) provides detailed three-dimensional vessel imaging invaluable for CTO PCI planning and guidance.

### 5.2. CCTA for Periprocedural Guidance in CTO PCI

CCTA has emerged as a unique tool for periprocedural guidance in CTO PCI, offering real-time co-registration capabilities in the catheterization laboratory. By importing CT datasets directly into the angiography system, interventionalists can benefit from continuous three-dimensional CT guidance throughout the procedure. This approach aligns CT reconstructions with C-arm angulation, providing a color-coded display of the coronary arteries in virtual CT and thus helping to identify fluoroscopic projections without foreshortening.

### 5.3. CCTA Co-Registration: Clinical Evidence

Research has shown that the CCTA co-registration technique can significantly improve CTO recanalization success rates. In a randomized study by Hong et al., patients undergoing a CTO PCI with CCTA guidance achieved better outcomes and had better procedural outcomes and lower complication rates than those receiving standard-of-care methods [43].

Recent advances in imaging technology explored the real-time fusion of three-dimensional CCTA with X-ray fluoroscopy for CTO PCI guidance. Studies by Ghoshhajra et al. and Xenogiannis et al. showed that this approach provides valuable insights into the vessel calcification, tortuosity, and proximal cap morphology, influencing the wiring strategy selection and potentially reducing the need for retrograde techniques [44,45]. Several studies have developed CT angiography-derived scoring systems to predict CTO PCI procedural success, as summarized in Table 2. While these initial observational studies are promising, larger trials are required to fully assess the impact of the CT/fluoroscopy fusion on CTO PCI.

### 5.4. CCTA Co-Registration: Implementation Challenges and Evidence Limitations

CCTA co-registration faces substantial implementation barriers limiting routine clinical applications in CTO interventions. The registration accuracy depends critically on patient positioning consistency and respiratory motion control, with potential misalignment errors that could misdirect the interventional strategy and compromise procedural outcomes. The CT integration adds significant procedural complexity, requiring specialized software, additional personnel training, and an extended setup time, questioning the applicability outside expert centers.

The evidence quality remains limited as most CCTA co-registration studies employ single-center observational designs with a potential selection bias. The Hong et al. randomized trial, despite positive results, enrolled only 240 patients at a single experienced center, significantly limiting the external validity. The combined CT and fluoroscopic exposure increases the cumulative radiation dose by 20–40%, raising patient safety concerns, particularly in failed procedures that require repeat attempts. Meanwhile, economic uncertainties persist, as CCTA co-registration (Philips HeartNavigator version 2.1) requires expensive specialized software licenses and high-end angiography systems, without supporting cost-effectiveness data to justify widespread adoption. Hardware requirements further limit the availability to advanced centers, potentially creating disparities in CTO care delivery between well-resourced academic institutions and community hospitals, thereby restricting patient access to this potentially beneficial technology.

## 6. Future Perspectives

Pre-procedural CCTA in CTO recanalization currently requires the standardization and optimization of radiation exposure and contrast volume protocols. Scheduling CCTA shortly before PCI has proven to be safe regarding contrast nephropathy. The field is advancing toward an innovative “one-stop-shop” approach that integrates CT perfusion with angiography [46]. This comprehensive strategy provides a simultaneous anatomical and functional assessment, enabling the detailed evaluation of the lesion morphology, vessel anatomy, and myocardial perfusion in a single session [47,52]. This approach warrants further research in the future. In addition, CCTA has emerged as a transformative tool in CTO management, offering clinicians unprecedented insights into complex anatomical features. Combined with periprocedural navigation, this advanced imaging modality shows significant promise in improving procedural success rates and efficiency. As technology continues to evolve, these non-invasive evaluation techniques are poised to revolutionize CTO treatment by enabling more precise and safer interventional approaches.

## 7. Critical Limitations and Implementation Barriers

Intravascular imaging in CTO interventions faces significant methodological and practical limitations that challenge widespread adoption. Most supporting studies are observational or small randomized trials (median n = 230 patients) conducted at expert centers, raising generalizability concerns for routine practice. A selection bias exists as studies typically exclude high-risk patients with renal dysfunction, severe calcification, or hemodynamic instability—precisely the populations where imaging guidance might provide the greatest benefit. The publication bias favors positive results, potentially overestimating clinical benefits, while most trials emphasize surrogate endpoints rather than patient-centered outcomes.

Technical limitations vary by modality: IVUS suffers from a limited calcification visualization and side-looking imaging requirements, OCT faces contrast-related safety concerns and limited tissue penetration (1–2 mm vs. 4–8 mm for IVUS), while the CCTA co-registration depends on positioning consistency and may increase the radiation exposure by 20–40%. Economic barriers include additional procedural costs (USD 500–1500 per case), extended procedure times (15–30 min), and infrastructure requirements for specialized equipment and software unavailable in many centers. Operator learning curves require substantial experience (typically 50+ cases for proficiency), creating quality disparities between high- and low-volume centers and potentially limiting benefits to experienced operators at well-equipped institutions.

## 8. Personalized Medicine Perspectives and Future Directions

Real-time imaging enables dynamic procedural adaptation through immediate strategy modification, wire selection adjustments, and personalized stenting decisions based on the encountered anatomy.The comparative advantages and disadvantages of each intravascular imaging modality in CTO PCI are summarized in Table 3.

Future Directions—Emerging technologies may enhance personalized CTO-PCI through real-time artificial intelligence for instant success prediction, live computational modeling for immediate strategy testing, and integrated physiological monitoring for dynamic procedural optimization. These advances could enable truly personalized interventions with a continuous adaptation based on live patient data.

## 9. Conclusions

Intraprocedural imaging enhances CTO-PCI through real-time guidance that improves technical success rates from 60% to 85–90% and reduces major adverse events, with IVUS-guided procedures demonstrating lower MACE rates (2.6% vs. 7.1%) and decreased stent thrombosis (0.9% vs. 6.1%) compared to conventional approaches. However, significant limitations persist, including small randomized trials with a selection bias, unclear cost-effectiveness given additional procedural costs (USD 500–1500 per case), and implementation barriers such as operator learning curves and equipment requirements that restrict widespread adoption. Large-scale randomized trials currently enrolling over 5000 patients (CRUISE-CTO, IMPROVE, IVUS-CHIP) will provide definitive evidence, while standardized protocols and cost-effectiveness analyses remain essential for optimal implementation. The current evidence supports selective rather than routine imaging use in CTO interventions, with the greatest benefit likely achieved in complex cases performed by experienced operators at well-equipped centers, and the future integration of artificial intelligence may address current limitations while expanding accessibility.

## Figures and Tables

**Table 1:** Ongoing trials are investigating the added value of intravascular imaging in CTO PCI.

Trial Name	Sample Size	Locations	Period	Primary Endpoint (s)	Secondary Endpoints	Key Inclusion Criteria	Study Design	Patient Population	Expected Clinical Impact
CRUISE-CTO	1448	China (45 centers)	2022–2031	MACE (death, MI, stent thrombosis, target vessel revascularization) at 12 months	Procedural success Procedural time Contrast volume Stent expansion	Symptomatic CAD At least one CTO lesion J-CTO score ≥ 2< Viable myocardium in CTO territory	Prospective, multicenter, open-label RCT	CTO lesions with moderate–high complexity	Establish IVUS-guided PCI as a standard of care for CTO Define optimal IVUS criteria for CTO intervention Potentially reduce long-term MACE rates
IMPROVE	2500–3100	US, Canada, Europe (120 centers)	2020–2027	1. Minimum stent area by IVUS 2. Target vessel failure at 12 months	Device-oriented composite endpoint Patient-reported outcomes Cost-effectiveness analysis	Complex coronary lesions ≥1 high-risk feature Suitable for PCI LVEF > 30%	Prospective, single-blind RCT	Complex lesions, including CTO, bifurcations, calcified lesions, and long lesions	Provide definitive evidence on imaging guidance for complex PCI Establish cost-effectiveness of routine IVUS Inform society guidelines on imaging use
IVUS-CHIP	2022	7 European countries (40 centers)	2021–2025	Target vessel failure (cardiac death, target vessel MI, target vessel revascularization) at 2 years	All-cause mortality Stent thrombosis Bleeding complications Quality of life measures	≥1 complex coronary lesion SYNTAX score ≥ 23 Vessel diameter ≥ 2.5 mm No contraindication to DAPT	Randomized, controlled, multicenter	Complex coronary lesions including calcified, ostial, bifurcation, left main, CTO	Define role of IVUS in high-risk PCI Potentially reduce need for repeat revascularization Guide optimal stent selection and deployment Influence European practice guidelines

Abbreviations: MACE: Major Adverse Cardiac Events; MI: Myocardial Infarction; CAD: Coronary Artery Disease; CTO: Chronic Total Occlusion; J-CTO: Japanese CTO score; IVUS: Intravascular Ultrasound; PCI: Percutaneous Coronary Intervention; LVEF: Left Ventricular Ejection Fraction; SYNTAX: Synergy Between PCI With Taxus and Cardiac Surgery; DAPT: Dual Antiplatelet Therapy; and RCT: Randomized Controlled Trial.

**Table 2:** Studies on computed tomography angiography-derived scores for prediction of chronic total occlusion (CTO) percutaneous coronary intervention.

Study Authors	Score Name	Design	Recruitment Period	Type of CT	No. of CTOs	Retrograde Approach	External Validation
Li et al. [48]	J-CTO CT	NR, retrospective	2011–2014	64-slice dual source	171	NR	Yes
Fujino et al. [49]	J-CTO CT	Single-center, retrospective	2012–2016	320-slice	218	33%	Yes
Li et al. [50]	RECHARGE CT	Multicenter, retrospective	2016–2019	64/128-slice dual source	367	28%	Yes
Yu et al. [51]	KCCT	Multicenter, retrospective	2007–2015	64-slice (including dual source)	456	12%	No
Opolski et al. [52]	CT-RECTOR	Multicenter, retrospective	2007–2013	64/128-slice dual source	240	11%	Yes

Abbreviations: NR: Not Reported.

**Table 3:** Comparative advantages and disadvantages of intravascular imaging modalities in CTO PCI.

Imaging Modality	Advantages	Disadvantages
**IVUS**	Superior tissue penetration (4–8 mm)	Limited resolution (100–150 μm)
	Excellent for vessel sizing and stent optimization	Cannot differentiate plaque subtypes well
	Real-time guidance for wire navigation	Side-looking imaging may miss some details
	Effective in heavily calcified lesions	Requires larger guide catheters
	No contrast requirement	Additional procedural time (15–30 min)
	Extensive evidence base in CTO PCI	Cost (USD 500–800 per case)
	Can guide both antegrade and retrograde approaches	Operator learning curve required
	Useful for proximal cap puncture guidance	May require balloon predilatation for insertion
**OCT**	Superior resolution (10–20 μm)	Limited tissue penetration (1–2 mm)
	Excellent plaque characterization	Requires contrast for blood clearance
	Optimal for stent deployment assessment	Safety concerns in renal dysfunction
	Superior detection of edge dissections	May exacerbate dissections
	Excellent for post-procedural evaluation	Limited evidence in CTO populations
	Can identify tissue coverage and apposition	Not suitable for large vessel assessment
	Useful for in-stent CTO characterization	Multiple contrast injections required
		Less useful during wire navigation
**CCTA Co-registration**	3D vessel visualization	Requires specialized software and equipment
	Excellent anatomical roadmap	Registration accuracy dependent on positioning
	Helps identify optimal projections	Increased radiation exposure (20–40%)
	Reduces foreshortening	High implementation costs
	Useful for complex anatomy navigation	Limited availability outside expert centers
	Can influence strategy selection	Potential misalignment errors
	Provides calcification assessment	Extended setup time
	Non-invasive pre-procedural planning	Limited real-time adaptability
